# The Evolution of Emerging Nanovesicle Technologies for Enhanced Delivery of Molecules into and across the Skin

**DOI:** 10.3390/pharmaceutics16020267

**Published:** 2024-02-13

**Authors:** Elka Touitou, Hiba Natsheh

**Affiliations:** The Institute for Drug Research, School of Pharmacy, Faculty of Medicine, The Hebrew University of Jerusalem, Ein Kerem, P.O. Box 12065, Jerusalem 9112102, Israel; hiba.natsheh@mail.huji.ac.il

**Keywords:** nanovesciles, soft, flexible, dermal/transdermal drug delivery, skin, ethosome, transfersome, niosome, glyecerosome, phospholipid vesicles

## Abstract

This review focuses on nanovesicular carriers for enhanced delivery of molecules into and across the skin, from their design to recent emerging technologies. During the last four decades, several approaches have been used aiming to design new nanovesicles, some of them by altering the properties of the classic phospholipid vesicle, the liposome. Phospholipid nanovesicular systems, including the phospholipid soft vesicles as well as the non-phospholipid vesicular carries, are reviewed. The altered nanovesicles have served in the manufacture of various cosmetic products and have been investigated and used for the treatment of a wide variety of skin conditions. The evolution and recent advances of these nanovesicular technologies are highlighted in this review.

## 1. Introduction

**Figure 1:**
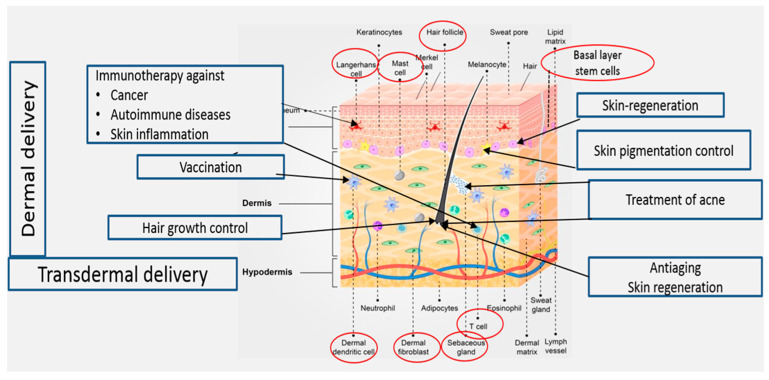
Treatments targeting cellular elements in the skin.

Delivery of drugs and active molecules into and across the skin for both local and systemic effects is an attractive field of research. Topical administration of pharmaceutical and cosmeceutical agents for the treatment of skin disorders may allow effective treatment of various skin ailments, including skin cancer, autoimmune diseases, skin inflammation, acne vulgaris, skin pigmentation, skin aging, hair loss, and skin regeneration. Treatment of these conditions requires targeted drug delivery to various skin regions (Figure 1). Transdermal drug delivery to systemic circulation offers several advantages, including the avoidance of side effects in the gastrointestinal tract. Moreover, using this delivery method, the hepatic first-pass metabolism can be avoided, leading to improved treatment outcomes [1].

As is known, the impermeable nature of the skin limits access to therapeutics through this route. Many approaches have been employed to overcome the skin barrier and facilitate drug delivery into and across the skin. Active and passive methods are used for the enhancement of skin penetration. Active methods include microneedles, electrophoresis, and sonophoresis. On the other hand, passive approaches focus on carrier-mediated delivery such as chemical permeation enhancers and nanovesicular carriers. Dosage forms based on nanovesicular carriers based on amphiphilic compounds have distinct advantages over conventional ones. These carriers can be classified into two major categories: phospholipid and non-phospholipid-based nanovesicles [2,3]. Transfersomes and ethosomes are the most investigated phospholipid nanovesicles for enhanced drug delivery into and through the skin. The two carriers evolved from liposomes that are obtained by the incorporation of surfactant edge activators and high concentrations of ethanol, respectively. These systems can also be tailored to prolong the drug effect and provide a controlled release of the active substance [4,5]. Surface-modified liposomes have also been investigated. One example is the AS1411-aptamer conjugated liposome. These present a new generation of liposomes that have been designed for safe and convenient delivery of 5-fluorouracil to the tumor site [6]. Niosomes represent the non-phospholipid category of nanovesicular carriers.

A previous review by Natsheh and Touitou [1] focused on the treatment of various skin conditions using phospholipid vesicles for dermal transdermal and nasal routes of administration. The authors emphasized the differences between a soft and an elastic phospholipid vesicle for dermal/transdermal and nasal drug administration. Lai et al. [7] reviewed various generations of phospholipid vesicular nanocarriers for application on the skin. In another interesting review in this field, Zoabi et al. [8] covered nanosystems including nanovesicles and other nanocarriers such as solid lipid nanoparticles and polymeric nanoparticles. Non-phospholipid nanovesicles are reviewed in publications focusing on the characterization, mechanism of action, and applications of these carriers [9,10]. The present analysis focuses on the evolution and emerging technologies based on nanovesicular carriers. Relevant work is covered including clinical applications and studies conducted in vitro, in vivo in animals, and clinically in humans.

## 2. Nanovesicles for Enhanced Drug Delivery into and across the Skin

**Figure 2:**
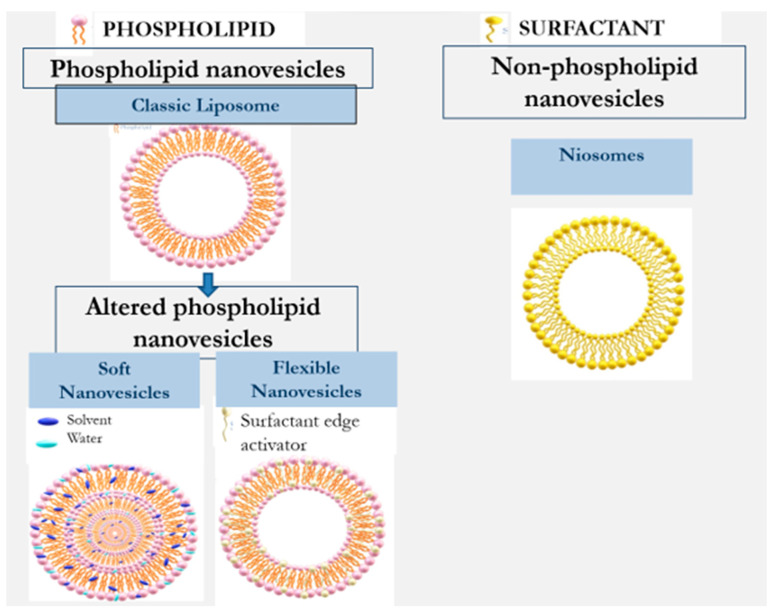
Evolution of nanovesicles for enhancement of dermal/transdermal drug delivery.

Nanovesilcular carriers for the design of formulations for application on the skin include liposomes, ethosome, transfersome, transethosmes, glycerosomes, and nisomes. They can be divided into two main types: phospholipid and non-phospholipid nanovesicles [2,11,12,13]. Table 1 and Figure 2 illustrate the evolution of nanovesicular carriers investigated for drug delivery into and across the skin.

### 2.1. Phospholipid-Based Nanovesicles for Dermal/Transdermal Drug Delivery

Liposomes are the classic and first investigated phospholipid vesicles. These are rigid nanovesicles, and their ability to enhance drug permeability to the deeper skin layers is limited. Several studies have indicated that these vesicles can carry drugs and active molecules only to the upper skin strata [11,12]. Further developed phospholipid carriers containing altered nanovesicles have been shown to overcome the permeability barrier in the skin [3,7]. The literature describes several approaches for altering the properties of conventional phospholipid vesicles to improve their penetration behavior. Transfersomes and ethosomes are the most investigated penetration-enhancing phospholipid nanovesicles. These nanovesicles are flexible and soft nanovesicles which are obtained by adding surfactant edge activators and alcohols, respectively [2]. These developments were followed by more recently designed transethosmes and glycerosomes. Niosomes are a non-phospholipid type of nanovesicles consisting mainly of non-ionic surfactants [13].

Cevc [15] introduced the transfersome as a modified liposome with flexible and elastic properties. Having a stress-responsive characteristic, these nanovesicles can penetrate skin openings much smaller than their own size. Their elasticity is imparted by the presence of an edge activator, generally a single-chain surfactant with a high radius of curvature. Once inserted between the phospholipids, the edge activator destabilizes the membrane bilayers, increasing their deformability. Sodium cholate, bile salts, oleic acid, Span 80, Tween 20, dipotassium glycyrrhizinate, and Tween 80 are examples of surfactants used for the preparation of transfersomes. Cevc used surfactants in transfersomal systems at a concentration range of 0.02–10% [19,20,21].

Deformable liposomes modified with edge activators or vegetable oils for improved dermal delivery of methotrexate have been investigated by Trotta et al. [22]. In this work, Epikuron 200 (phospholipid containing 95% phosphatidylcholine, PC) or hydrogenated lecithin (PL100H)-based liposomes were modified by adding the natural surface-acting agent dipotassium glycyrrhizinate (KG). These modified liposomes exhibited high deformability and elasticity, allowing them to pass through barriers with pores smaller than their own diameter by a factor of about three. The authors reported a similar size of these deformable liposomes before and after passing through 100 nm pores. Nanovesicles with a mean diameter of 352 ± 28 nm displayed a value of 345 ± 20 nm after the filtration, indicating their high degree of elasticity. The effect of these deformable liposomes on the skin permeation of methotrexate was evaluated in vitro on porcine ear skin. The cumulative methotrexate amounts permeated through the skin during 24 h from the deformable vesicles containing PC and PL100H were 23.55 ± 4.3 and 16.8 ± 4.0 µg, respectively. On the other hand, the rigid liposome and aqueous solution of the drug had lower permeation profiles of 5.7 µg. Zheng et al. [23] studied the morphology of soybean lecithin transfersomes modified with sodium deoxycholate containing itraconazole as an active compound model. Stable nanovesicles, composed of 6 mmol/L phospholipids, 6 mmol/L sodium deoxycholate, and 1.23 mmol/L itraconazole, were prepared using the thin layer evaporation method. The reported mean size of the obtained nanovesicles was 100 nm. Transmission microscopic examination indicated that these nanovesicles are spherical, uni-lamellar hollow associates.

**Figure 3:**
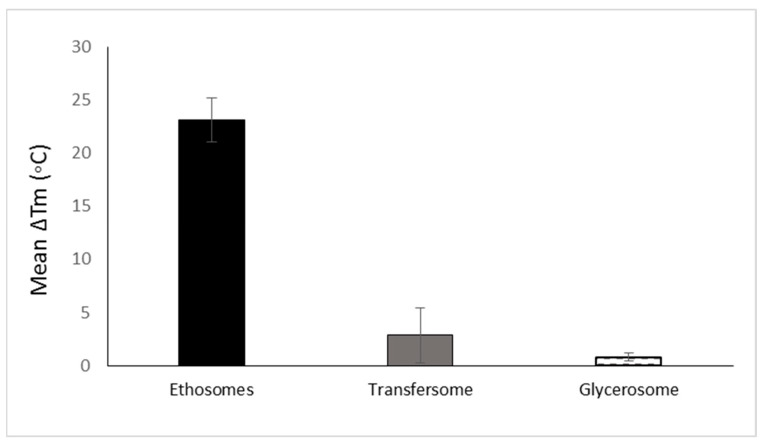
The mean reduction of Tm of various phospholipid vesicular carriers for trans-mucosal drug delivery dermal/transdermal as compared to the classic liposome based on data collected and published by Natsheh and Touitou [2].

The development of soft vesicles presents an interesting evolution of phospholipid vesicles. This type of nanovesicle is obtained owing to the presence of solvents such as alcohols. The first and most investigated soft vesicle is the ethosome, introduced by Touitou [12]. This nanovesicular carrier is composed of phospholipid, ethanol (20–50% *w*/*w*) with or without glycols, water, and the active molecule. Generally, ethosomes are multilamellar nanovesicles containing bilayers from wall to wall. The ethosomal phospholipid vesicles contain short-chain alcohols like ethanol or isopropyl alcohol with an optional addition of glycols such as Transcutol^®^ or propylene glycol. Phosphorous (31P) NMR data indicated that the high alcohol concentration in ethosomes imparts fluidity to the phospholipid bilayers and organizes them in a spherical lamellar-closed shape. This notable fluidity of the lipid bilayers in ethosomal vesicles, compared to liposome, was further confirmed by the results of fluorescent anisotropy measurements of 9-antrivinyl labeled analogue of PC (AVPC) and differential scanning calorimetry (DSC) data. A reduction of up to 30 °C was reported in the main Tm of the phospholipid in ethosomal nanovesicles when compared with the corresponding liposomes [11]. The changes in the Tm values ethosomes and other phospholipid nanovesicles are presented in Figure 3.

When comparing the effect of ethanol on the vesicle fluidity with that of surfactant edge activators, a significant difference can be observed. El Maghraby et al. showed that the addition of edge activators such as oleic acid, Tween 80, and Span 80 to dipalmitoylglycerophosphatidylcholine (DPPC) nanovesicles reduced the Tm values by 2.1, 0.84, and 7.3 °C, respectively. Trotta et al. [22] showed that PL100H vesicles containing 1% *w*/*w* KG had a transition temperature (Tm) value reduced by 0.9 °C in comparison with liposomes. It can be noted that the effect of solvents on vesicle fluidity is more significant than that of edge activators. This behavior is supported by the reduction in Tm values for nasally administered phospholipid nanovesicles modified by ethanol and propylene glycol, where the Tm was reduced by up to 16 °C (Figure 3).

Another distinguishing property of ethosome is the multilamellar structure from wall-to-wall that has been seen by electron microscopic (EM) micrographs. This structure of ethosomes was retained in drug delivery systems containing many drugs, such as minoxidil and testosterone [11,24]. On the other hand, the incorporation of other molecules such as buspirone and erythromycin yielded uni-lamellar ethosomes [4,25]. The reported dynamic light scattering data indicated a mean size distribution of 150 ± 4 nm for drug-free ethosomes composed of 2% phospholipid and 30% ethanol. Increasing the ethanol concentration from 20 to 45% led to a vesicular size reduction to 103 nm. It is worth mentioning that the corresponding liposomes, prepared by the conventional film-forming method, had a larger average size of 677 ± 31 nm [11].

The content and structure of the vesicles affect their properties, such as their capacity to incorporate the active molecule. As an example, the classic liposome vesicle contains an aqueous core surrounded by phospholipid lamella. The incorporated molecule, if soluble in water, will be dissolved in the core of the liposome. On the other hand, lipophilic molecules will be incorporated between the phospholipid bilayers. Different from the above structure, the ethosome contains phospholipid bilayers from wall-to-wall incorporating the ethanol and water between them. This unique structure enables the incorporation of both lipophilic and hydrophilic molecules in the whole vesicles.

Published data on ethosomes indicates their efficiency in delivering active molecules into and across the skin, in terms of quantity and depth, as compared to classic liposomes or a hydroalcoholic solution. This property was reported from an in vitro experiment using Franz diffusion cells and confocal laser scanning (CLS) microscopic examination, where the ethosomal system containing the fluorescence probe, calcein, penetrated mouse skin into a depth of 160 µm. This is in comparison with a hydroethanolic solution and liposome, which carried the molecule only to depths of 80 and 60 µm, respectively [11].

The extensive research published on transfersomes and ethosomes led to the development of a new generation of nanovesicular carriers including transethosomes, glycerosomes, and plurethosomes. Transethosomes are phospholipid nanovesicular carriers combining the properties of ethosomes and transfersomes by consisting of high ethanol content (≥20%) together with edge activators. Song et al. [17] prepared voriconazole transethosomes by adding the edge activators Tween 80, sodium taurocholate, and oleic acid at a concentration of 0.53% *w*/*w*, to ethosomes containing 30% ethanol. TE microscopic examination indicated the presence of irregular-shaped vesicles with a mean size in a range of 146 to 192 nm. These irregular morphological features could be a result of combining ethanol and edge activators that may rearrange the lipid bilayers of this type of vesicle. Rady et al. [26] prepared a Fe-chlorophyllin transethosomal system based on phosphatidylcholine, the edge activators Tween 80, Tween 20, Span-20 or Cremophor-A25, and 20% *w*/*v* ethanol. The reported mean vesicular size ranged between 455 to 686 nm when these surface-active agents were used at the 0.1% concentration. The values decreased to 305–385 nm when a higher concentration of edge activators was used (0.3% *w*/*v*). The authors also reported that vesicles containing 0.1% *w*/*v* Cremophor A25 exhibited a deformability index of 26.2 ± 3.8 mL/s.

Glycerosomes are based on another modification of phospholipid vesicles, containing glycerol at a concertation of 10–30% *v*/*v* [16]. The authors reported that this trihydric alcohol increases the fluidity of the vesicles’ bilayer, thus improving the ability of the carrier to enhance molecule penetration into the skin. However, the effect of glycerol on Tm values is considered low when compared to that of ethanol in ethosomes. The reduction in Tm due to the presence of glycerol in glycerosomes is quite small and does not exceed 0.9 °C. To understand the reason behind this minimal effect of glycerol on the vesicles fluidity, it should be noted that glycerosomes were prepared using DPPC, hydrogenated soy phosphatidyl choline (PL90H) and 1,2-dimyristoyl-sn-glycero-3 phosphatidylcholine (DMPC) [16,27,28]. In addition, these vesicles contain cholesterol, a membrane stabilizer, contributing to the vesicular rigidity [29]. TEM examination revealed that glycerosomes are multilamellar spherical nanovesicles with a mean diameter of 105–130 nm. The authors reported that the vesicle elasticity improved as glycerol content increased; empty and drug-loaded glycerosomes with 30% glycerol were two-fold more deformable than the control. Glycerosomal systems containing 20% and 30% glycerol were more efficient in delivering diclofenac into the skin compared to systems containing 10% glycerol or conventional liposomes.

### 2.2. Non-Phospholipid Nanovesicles

Nanocarriers based on non-phospholipid materials are another development of vesicles for dermal and transdermal applications. These include solid lipid nanoparticles and nanostructured lipid carriers, polymeric nanoparticles, and the well-known non-phospholipid nanovesicular carriers, niosomes [8].

Niosomes are nanovesicles made of non-ionic surfactants, unlike all the upper discussed carriers containing phospholipid as a vesicle-generating amphiphile. They are liposome-like vesicles consisting of hydrated mixtures of nonionic surfactants such as mono- or dialkyl polyoxyethylene ether and cholesterol. These nanovesicular carriers have been developed as alternative controlled drug delivery systems aiming to overcome the problems of liposomes related to large-scale production, sterilization, and stability [9]. Niosomes are self-assembled nanovesicles and are obtained by the hydration of synthetic surfactants and appropriate amounts of cholesterol. The first niosomal formulations were developed and patented by L’Oreal in 1975 for cosmetic purposes. Thermodynamically stable niosomes can be only obtained when using mixtures of surfactants and charge-inducing agents at a proper concentration [3]. Microscopically, niosomes appear as multilamellar or unilamellar closed nanovesicles. One of the important evolutional steps in the development of niosomes was the introduction of detergent (Tween 20, HLB value = 16.7) based niosomes by Santucci et al. by the end of the 1990s [30].

More recently, niosomes acquired growing attention as promising drug delivery systems. Marianecci et al. [31] designed niosomes based on Tween 85, Span 20, and cholesterol for enhanced delivery of ammonium glycyrrhizinate (AG), for the treatment of skin inflammation. TE micrographs of the niosomal vesicles loaded with AG indicated that these are unilamellar spherical closed vesicles. DLS measurements indicated that the drug-loaded niosomes possess a mean vesicular size of around 100 nm and PDI values in a range of 0.3. As stated by the authors, the addition of cholesterol to niosomes plays a fundamental role in vesicle formation increasing the bilayer’s rigidity. Niosome samples lacking this ingredient showed a high fluidity in vesicle bilayers. Interestingly, cholesterol is a bilayer anti-fluidizing agent.

One of the important parameters affecting the properties of niosomes in the preparation method. They can be prepared by a number of methods. The ether injection method is based on dissolving the ingredients in ether, which is then slowly injected into an aqueous medium at a high temperature. The resulting niosomes are large unilamellar vesicles (LUV) [30]. Multilamellar vesicles (MLV) can be obtained using the film method, where the mixture of surfactant and cholesterol is dissolved in an organic solvent which is then removed by vacuum at room temperature. The resultant dry surfactant film is then hydrated by agitation at 50–60 °C [32,33]. Another interesting method is the sonication of a mixture containing the aqueous phase, surfactants, and cholesterol. The obtained vesicles are small unilamellar (SUV) niosomes [34].

## 3. Mechanism of Skin Penetration Enhancement via Nanovesicular Carriers

Multiple studies aimed to investigate the mechanism of action of the emerging nanovesicular carriers to explain their superiority over conventional rigid liposomes which remain stuck in the upper stratum layers. Being soft or flexible, these carriers act via multiple mechanisms to enhance molecule penetration into and across the skin.

As mentioned above, soft nanovesicles contain high solvent concentration which fluidizes the vesicular bilayer and disrupts the lipid bilayers in the SC. These changes facilitate the penetration of the vesicles deeper into the skin, where their active content is released [1,2].

**Figure 4:**
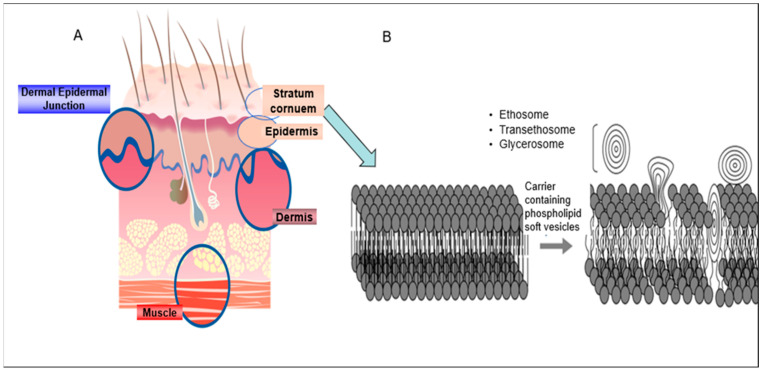
(**A**) skin structure; (**B**) proposed mechanism of action of soft nanovesicular carriers: fluidization of the lipid bilayers in the SC and opening the “door” for the penetration of the active molecules into the deep skin layers. The penetration enhancement is a result of the action of the nanovesicular system reproduced from [1] with permission.

The high ethanol concentration (up to 50%) in the composition imparts two properties during the drug delivery enhancement process, fluidizing the phospholipid bilayers in the vesicles and disrupting the lipid organization in the SC (Figure 4). As a result, vesicles are allowed to penetrate the deep skin layers leading to enhanced dermal/transdermal drug delivery in comparison with the rigid liposomes. This model was first introduced by Touitou et al. [11], who also suggested that ethosomes enhance molecule penetration through the skin mainly via the intracellular pathway. The authors supported this assumption by the results of enhanced in vitro delivery of several molecules with various properties into fibroblasts. In this study, the behavior of the amphipathic probe 4-(4-diethylamino) styryl-N-methylpyridinium iodide (D-289), the lipophilic probe rhodamine red (RR), and fluorescent phosphatidylcholine (PC *) was assessed by confocal laser scanning microscopy (CLSM) and fluorescence-activated cell sorting (FACS). The fluorescence intensity of RR by measured CLSM was found to be 150, 40, and 20 arbitrary units (A.U.) for the probe delivered in ethosomal carrier, hydroethanolic solution, and corresponding liposomes, respectively. FACS results pointed towards the same trend where a visible penetration of the incorporated probes was observed only for ethosomes.

The above model was confirmed by many studies. Mbah et al. [35] investigated the effect of an ethosomal gel containing griseofulvin on the Tm of excised rat skin. The DSC results indicated the presence of a reversible endothermic peak at 69.9 °C which turned to an enlarged peak at 94.0 °C. This was explained as a consequence of the SC fatty acids fluidization.

Cevc and Blume proposed a different mechanism of percutaneous permeation enhancement for transfersomes. They hypothesized that the difference in total water concentration between the skin surface and its deeper layers creates an osmotic gradient enforcing the vesicles to flow into and through this barrier. Interestingly, these driving forces were shown to be able to push more than 0.5 mg of transfersomes per hour and cm^2^ through the SC but not into the deeper skin layers. Previous studies reported the dependence of transfersomes efficiency, as penetration enhancing nanocarriers, on non-occlusive skin application. This mode of application is crucial to allow water evaporation, and the creation of a hydration gradient to enable penetration of great quantities of applied lipids into the deeper skin layers. Cevc and Blum [15] reported that non-occlusive application of tranfersome led to skin permeation of more than 50% of the total Tritium-labelled 1.2-dipalmitoyl.glycem.sn-phospho[N.mcthyl-3H]choline (3H-DPPC) applied amount. This is in contrast to occlusive application, where 87% of the lipid applied amount was confined to the skin surface.

**Figure 5:**
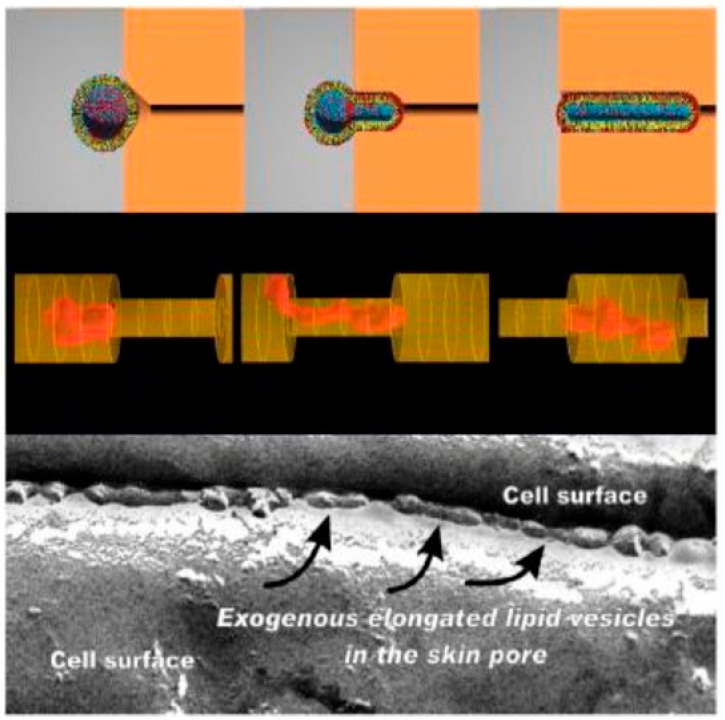
Proposed mechanism of skin penetration by transfersomes. (**Top**): computer-simulated distribution of more (red, e.g., a surfactant) or less (blue, e.g., a phospholipid) water-soluble molecules with hydrophobic (yellow) chains arranged in a mixed amphipathic spherical bilayer as a function of predefined vesicle shape. (**Middle**): a simulation of a highly deformable, infinitely permeable, non-destructible vesicle forced by a horizontal gradient into a pore with 0.5 smaller diameters. (**Bottom**): an electromicrograph of elongated, deformable vesicles in an inter-corneocyte water-filled channel within the human stratum corneum following the application of a lipid preparation on its open surface. Reproduced from Reference [37] with permission.

Schätzlein and Cevc [19] suggested two different hydrophilic pathways of delivery enhancement mechanism by means of tranfersomes: the tortuous intercluster route between groups of corneocytes and the intercorneocyte pathway passing between the individual corneocytes within the cell clusters. The flexible and elastic properties of transfersomes enable them to adjust their shape and penetrate the small pores in the dense SC layer (Figure 5) [36,37].

The mechanism of action of transethosomes as suggested by Song et al. [17] is based on a synergistic effect of ethanol and edge activators.

On the other hand, the action of glycerosomes involves the fluidization of the lipid bilayers in the skin as reported by Moolakkadath et al. [38]. DSC thermogram of rat skin treated with glycerosomes loaded with the fisetin in vitro indicated a change in the spectrum when compared to normal untreated skin and the disappearance of a Tm peak at 45.12 °C. According to the authors, this change resulted from structural changes in the skin due to lipid disruption and reorientation leading to increased fluidity of SC lipids. It was hypothesized that the modifying agent, glycerol, present in glycerosomes induced hydration of the skin via interaction with the polar groups of the lipids increasing their fluidity and facilitating the diffusion of the nanovesicles.

Modifying the phospholipid nanovesicles with glycerol minimally lowered the Tm of phospholipid bilayers. On the other hand, the deformability index of these vesicles was increased by at least 10%. Therefore, the mechanism of penetration enhancement of these vesicles may involve their deformability and elasticity rather than their softness [2].

**Figure 6:**
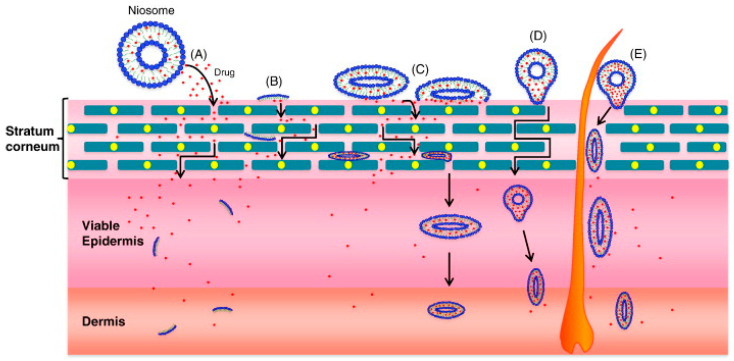
Proposed mechanisms of action of niosomes for dermal and transdermal applications: (**A**) drug molecules are released by niosomes; (**B**) niosome constituents act as penetration enhancers; (**C**) niosome adsorption and/or fusion with Stratum Corneum; (**D**) intact niosome penetration through the intact skin; (**E**) niosome penetration through hair follicles and/or pilosebaceous units. Reproduced from [9] with permission.

Niosomal-based systems are reported to enhance skin permeation in a sustained release mode as compared to liposomes. Two mechanisms are proposed for the permeation enhancement of drugs loaded in these nanovesicles: the permeation-enhancing effect of components and the direct vesicle fusion with the SC (Figure 6) [9]. This theory was supported by several authors. As an example, Fang et al. [39] showed that the effect of niosomes on the transdermal administration of enoxacin was greater than that of liposomes.

From the above, each type of nanovesicle enhances drug delivery into and across the skin by a different mechanism. Briefly, high ethanol content in ethosomal nanovesicles contributes to their softness and the fluidity of the lipids in the stratum corneum facilitating the vesicle passage deeper into the skin. On the other hand, edge activators impart deformable and elastic properties to transfersomes, allowing them to pass through pores much smaller than their own size. Glycerosomes and transethosomes seem to combine the delivery mechanism of both flexible and soft nanovesicles.

## 4. Examples of Studies Carried out with Nanovesicles for Enhanced Drug Delivery

As a result of the significant attention given to nanovesicles as carriers in dermal and transdermal delivery research, numerous works have been published. This section reviews many of these publications categorizing them into in vitro, in vivo preclinical, and in vivo clinical studies. Being the most investigated nanovesicles to deliver active molecules into and across the skin, transfersomes, ethosomes, transethosomes, glycerosomes, and niosomes are discussed in the following sections.

### 4.1. In Vitro Studies

One important step during the design and investigation of novel drug delivery systems is the in vitro study. This is an indispensable tool to evaluate the effect of the nanovesicles on the permeation and penetration behavior of active molecules and probes. Table 2 summarizes some of these studies presenting the active molecules, the carriers, the methodology, and the results.

One of the first in vitro studies on soft nanovesicles was published by Touitou et al. [11] on skin permeation of minoxidil. The experiment was carried out on excised nude mice skin over 24 h. The ethosomal system applied non-exclusively improved the permeated amount of the drug by up to 45 times in comparison to the hydroethanolic solution. Trotta et al. [22] studied the effect of deformable liposomes on the 24 h skin permeation profile of methotrexate on pig ear skin. Systems containing PL100H and PC permeated cumulative drug amounts of 16.8 ± 4.0 and 23.55 ± 4.3 µg, respectively. These amounts were 4-fold higher than permeated by classic liposome and aqueous solution.

Another interesting in vitro study by Paolino et al. [41] aimed to examine the anti-tumor effect of the paclitaxel ethosomal system on the human squamous-cell-carcinoma line (DJM-1) in comparison with the physical mixture of the drug and the carrier. The reported results indicated an in vitro percutaneous drug permeation profile of 103.5 µg/cm^2^ upon system 24 h application as compared to 20.35 and 4.31 µg/cm^2^ for the physical mixture and the hydroethanolic solution, respectively. Furthermore, the paclitaxel ethosomal system exhibited an efficient anti-proliferative activity in DJM-1 cells leading to cellular mortality of ~40% of the cultures after 48 h. This result presented an improvement of ~2.2-fold compared to the effect of the drug in the physical mixture at the same drug concentration.

In an in vitro study on fibroblasts and 3D-reconstituted human epidermis, the antioxidant effect of coenzyme Q10 incorporated in ethosomes was evaluated. The reported results indicated that pretreatment of the cells with the ethosomal system counteracted the H_2_O_2_-induced oxidative stress and prevented the formation of the oxidative damage biomarker 4-hydroxynonenal protein. The authors also reported enhanced antioxidant activity of Coenzyme Q10 in primary dermal fibroblasts by 35% by means of the ethosomal system [47].

Currently, quercetin is under investigation for the treatment of several skin pathologies, including skin cancer. Ethosomes and transethosomes containing this molecule were investigated in vitro on melanoma cells by Ferrara et al. [40]. Quercetin is a natural flavonoid with proven anti-inflammatory, antioxidant, antiviral, antimicrobial, and anticarcinogenic properties. Permeation studies on excised human skin indicated that quercetin solution displayed the lowest permeability coefficient values (10-fold and 6-fold lower with respect to transethosomes and ethosomes, respectively (*p* < 0.0001). Notably, scratch wound tests perfumed on confluent monolayer of HaCaT cells and melanoma HT-144 cells indicated that quercetin nanovesicular systems enabled a more efficient wound closure suggesting their potential use as adjuvant strategy for such skin conditions.

Plurethosome in a modified ethosomal carrier containing the block copolymer pluronic. Sguizzato et al. [18] designed plurethosomes containing maginferin and investigated their antioxidant and anti-inflammatory effects against pollutants on 3D human skin models exposed to O_3_. In this model, exposure to O_3_ leads to a significant increase in 4HNE protein adduct levels compared to untreated tissues exposed to air. The published results indicated that topical administration of the nanovesicular system reduced the production of 4HNE levels by 2-fold, exerting protection against oxidative stress. Micrographs of immunofluorescence staining of the 3D skin models indicated that plurethosomes enabled efficient delivery of magniferin into the deeper layers of the skin. This effect has been maintained even 24 h after O_3_ exposure, indicating the vesicle’s capability to retain the drug and prolong its release.

Gel-embedded niosomes were also investigated by Coviello et al. [48]. The vesicles, composed of Tween 20 and cholesterol or of Tween 85 and Span 20, and loaded with monoammonium glycyrrhizinate were embedded in Xanthan and Locust Bean Gum matrices. Results of the in vitro release studies showed a slower diffusion rate of the active molecule loaded in niosomes and embedded in the gum matrices as compared to non-embedded vesicles.

### 4.2. In Vivo Preclinical Studies

During the last four decades, numerous in vivo studies have been carried out to investigate the ability of nanovesicles to facilitate the delivery of active molecules into and across the skin. These include pharmacokinetic and pharmacodynamic studies on animal models of induced skin and systemic diseases. Preclinical studies in animal models for a specific disease can be a stage in translational studies. Skin inflammation, psoriasis, skin cancer, acne vulgaris, hair loss, and skin aging are the most investigated skin medical conditions. Diabetes, hypertension, and hot flashes are examples of systemic diseases that have been induced in animal models to study the effect of the transdermal application of drugs in nanovesicles. These in vivo preclinical studies are presented in Table 3.

CBD is extensively investigated due to its promising applications. Touitou’s group incorporated this molecule in ethosomes and studied the pharmacokinetic and pharmacodynamic effect of the system on animal models of induced arthritis. The reported results indicated that CBD steady-state levels were achieved following a 24 h application to mice abdominal skin and lasted at least to the end of the 72 h experiment. In addition, ethosomal CBD prevented inflammation and edema in mice with carrageenan-induced inflammation [53].

Efficient treatment of deep skin bacterial infections in mice by erythromycin ethosomes was reported by Touitou’s group [25]. In this study, the ethosomal system applied to ICR mice skin, previously inoculated with *S. aureus* ATCC29213, completely inhibited the infection. The outcomes of this study show that ethosomes are efficient carriers of antibiotics for the treatment of deep skin infections. This is in contrast with the treatment with erythromycin hydroethanolic solution where deep dermal and subcutaneous abscesses developed showing the limited ability of the solution to carry the drug to the site of action.

Another interesting achievement in the field of in vivo research on transdermal drug delivery via nanovesicles is the efficient management of hot flushes in animal models by buspirone ethosomal systems [4]. Transdermal administration of the formulation enabled the delivery of buspirone to plasma over a 12 h period, with a Cmax value of 120.07 ± 86.97 ng/mL after 2 h. A relative bioavailability of 0.89 was calculated for transdermal drug administration vs. oral. The ability of the transdermal Buspirone ethosomal system to alleviate the temperature rise in hot flushes was evaluated in ovariectomized (OVX) rats in comparison to oral administration of the drug. The obtained results indicated that 3 h following administration of buspirone transdermal system, a reduction of 1.6 ± 0.7 °C in the tail skin temperature (TST) was observed. These values are considered normal values and last 3 h.

NSAIDS are one of the pharmacological drug categories that present promising candidates for delivery into and across the skin via nanovesicular carriers. Shumilov et al. studied the antipyretic effect of the Ibuprofen ethosomal system in a rat model of brewer yeast-induced fever. Treatment with an ethosomal Ibuprofen system led to temperature alleviation to normal values that lasted at least 12 h versus only 7 h after the oral treatment [2]. In another study, El-Menshawe et al. [60] measured the anti-inflammatory effect of Meloxicam niosomes using the carrageenan-induced rat paw edema method in rats. Vesicles composed of sorbitan monostearate (Span 60), and cholesterol (CH), were prepared by the thin film hydration method. Results of the in vivo study indicated that the paw edema was reduced by 2 times in comparison with conventional gel.

An interesting study was conducted with transfersomal gel containing adapalene and vitamin C for the treatment of acne vulgaris in a rat model of testosterone-induced acne [52]. Testosterone application for four weeks produced 50 ± 11 papules. Treatment with adapalene and vitamin C transfersomal system reduced the papule count to 14 ± 8. Counts of 17 ± 7 and 24 ± 9 papules were reported for transfersome lacking the vitamin and for the group treated with the commercial gel, respectively.

Transfersomes containing centrally acting drugs present an interesting field of research. Gupta et al. [50] evaluated the ability of transfersomes to enhance the transdermal delivery of poorly soluble anti-psychotic drug, sertraline. In vivo studies on mice models of psychosis using the swim force test, revealed that the transfersomal gel exhibited better antidepressant activity with a minimal immobility time of 0.323 min compared to 2 min in the mice that received the conventional sertraline gel. Asenapine maleate is another antipsychotic drug that has been incorporated into transethosomes to improve its bioavailability. Shreya et al. [56] studied the pharmacokinetic profile of topically applied to rats’ skin asenapine transfersomal carrier modified with 20% *v*/*v* ethanol. The reported results indicated a significant (*p* < 0.05) increase in the bioavailability following transdermal application of the transdermal system compared with oral administration.

Albash et al. [55] investigated the anti-hypertensive effect of an olmesartan medoxomil transethosomal system in a rat model of elevated blood pressure. The effect of the system was compared to that of an orally administrated commercial tablet. Hypertensive blood pressure values above 150 mmHg were obtained by subcutaneous injection of methylprednisolone acetate. The reported data indicated that the transdermal treatment normalized the blood pressure values for 24 h, with the highest percentage reduction at four hours (35 ± 12%). On the other hand, oral drug administration led to desired blood pressure values for six hours only.

### 4.3. Clinical Studies

Clinical studies are requested to prove the efficacy of new treatments and drugs. Starting from the 90s, a considerable part of the research on nanovesicular-based formulations focused on the clinical examination of their safety and efficacy. Table 4 summarizes some of these studies.

One early study on healthy volunteers examined the hypoglycemic effect of transfersomal insulin as compared to a subcutaneous injection [5]. Insulin formulations were administered to the inner forearm at a dosage of 0.6 U/kg. As documented by the researchers, the application of the insulin transfersomal system produced a prolonged hypoglycemic response. The transfersulin formulation exhibited an onset of action between 90 and 180 min, which is 45–145 min longer than that observed with the subcutaneous insulin administration.

In a two-armed, double-blind, randomized clinical study on 40 participants with Herpes labialis, the anti-viral effect of acyclovir ethosomes was tested. The efficacy of the new treatment was evaluated as compared to the commercial cream (Zovirax^®^, GlaxoSmithKline Manufacturing S.p.A, Parma, Italy) and to empty ethosomes. In one arm of the clinical evaluation, the documented lesions’ crusting time was 1.6 in the group treated with ethosomal acyclovir vs. 4.3 and 4.8 days for the groups treated with a commercial product and an empty vehicle, respectively. A similar trend of the results was obtained in the crossover arm. A significant reduction in the proportion of abortive lesions was reported in 30% of episodes treated with ethosomes in the two arms. This percentage presents a 3-fold improvement as compared to the group treated with Zovirax [66].

**Figure 7:**
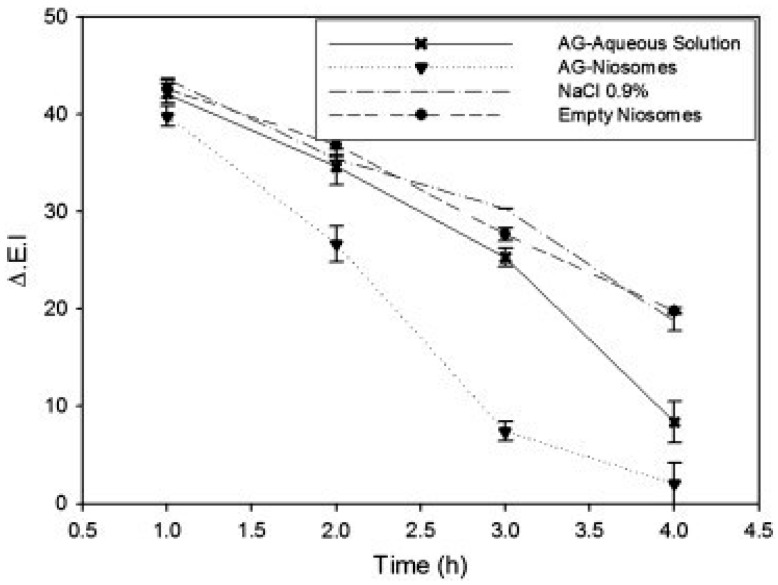
In vivo anti-inflammatory activity in human volunteers of niosomes containing ammonium glycyrrhizinate evaluated as the ability to reduce a skin erythema, chemically induced by pretreatment with an aqueous solution of methyl-nicotinate. Reproduced from [31] with permission.

Ammonium glycyrrhizinate is a natural anti-inflammatory agent that has been incorporated into various nanovesicles including ethosomes, niosomes, and ultra-deformable liposomes. These interesting studies were published by Paolino’s group as three clinical trials on healthy volunteers with methyl nicotinate-induced skin erythema. Ethosomal system containing ammonium glycyrrhizinate was evaluated on 12 human volunteers. The erythema index (ΔEI), measured using a reflectance visible spectrophotometer, was 29.6% for the ethosomal system versus 62.7 and 60.7% for the comparative ethanolic and aqueous solutions, respectively [65]. In another two works, treatment with niosomes and ultra-deformable liposomes reduced the erythema index by 3.4- and 15-fold, respectively, when compared to an aqueous solution (Figure 7) [31,63].

In an 8-week randomized double-blind trial on 40 patients by Touitou et al. [2], efficient treatment of acne vulgaris was achieved by incorporating clindamycin and salicylic acid into ethosomal gel. The reported results revealed an improvement in the condition of 71% of the participants. In addition, eighty percent of the participants with a history of previous topical treatments stated a superior tolerability of the ethosomal gel which caused less erythema, burning, pruritus, and photosensitivity reactions compared to prior topical medications such as clindamycin lotion or erythromycin solution.

Another 12-week study conducted on knee osteoarthritis (OA) patients (*n* = 866) showed the efficacy of the ketoprofen transfersomal system when evaluated in comparison with a ketoprofen-free vehicle. Patients were divided into four random groups and treated twice daily with the topical transfersomal system containing 25, 50, or 100 mg ketoprofen, or with the control-free vehicle. The pain expression was reduced by 88.6, 86.8 and 88.6% for the three-drug doses applied in transfersomes, respectively. On the other hand, the control treatment alleviated the pain expression by 77.5% [64].

One study investigated griseofulvin incorporated in niosomal gel and examined sixteen patients with tinea circinata in comparison to placebo and a liposomal gel of the drug. Tinea corporis infections are common fungal skin infections caused by *Epidermophyton floccosum*, Trichophyton, and Microsporum species. Clinical and mycological evaluations after 3 weeks indicated a significant improvement in the participant groups treated with the drug in the niosomal and liposomal systems. Mild and transient irritation was reported in 4 participants [69].

A clinical study on finasteride applied topically in niosomal and proniosomal systems consisting of cholesterol and Span 80 for the treatment of androgenetic alopecia was carried out by Vimal et al. [70]. Clinical evaluation by phototrichogram method was performed for proniosomal formulation on twenty healthy male volunteers. The outcome of this study indicated an increase in the hair count by 42.85% when compared with the control group (6.6%).

In another controlled clinical experiment by Manosroi, et al. [71] the skin anti-aging effect of niosomal gel loaded with gallic acid extracted from *Terminalia chebula* galls was studied on healthy volunteers. This plant appears in many Thai Lanna medicinal recipes for the promotion of longevity. The formulations consisted of nanovesicles containing Tween 61 mixed with cholesterol. The skin elasticity and roughness were monitored using a Cutometer (Courage & Khazaka, Cologne, Germany) upon system application and treated twice a day for 8 weeks. The percentage changes of the two parameters, skin elastic recovery, and skin elastic extension, when two niosomal formulations were applied, were +28.73 and +32.57; −21.25 and −22.63%, respectively. A significant decrease in the maximum and average roughness values with the parameter changes being −29.43 and −32.38, −39.47 and −35.28%, respectively.

Manosroi, et al. [72] measured the anti-aging efficacy of topical formulations containing niosomes entrapping semi-purified rice bran bioactive compounds on rabbits and 30 human volunteers. The skin hydration, elasticity, thickness, roughness, and pigmentation were measured by corneometer, cutometer, visiometer, and mexameter, respectively. The extract entrapped in niosomes did not cause erythema or edema within 72 h on shaved rabbit skin by the closed patch test investigated by a mexameter and visual observation, respectively. Application of the compositions to the skin of the human volunteers, for 28 days, led to improvement of skin hydration, lightening, thickness, roughness, and elasticity by 9, 27, 7, 3, and 3 times, respectively.

All the nanovesicular systems discussed above can be suitable for pharmaceutical and cosmetic products. When choosing a nanovesicular carrier for incorporating a molecule for a specific application, the formulator should be aware of the components of the nanovesicular system and its compatibility with the active ingredients as well as their indication. As an example, compatibility of the active ingredient with ethanol is an important requirement for a stable ethosome formulation. Furthermore, when looking for a carrier for a molecule active on the upper layers of the skin, the liposome is a good candidate, while an altered vesicle is more adequate for the delivery into the deeper layers or transdermal absorption.

## 5. Biocompatibility of Nanovesicles for Dermal and Transdermal Delivery

The tolerability and biocompatibility of various nanovesicular systems were tested in safety experiments in in vitro, in vivo, and clinical studies [2]. Some representative examples are presented below.

In one clinical study carried out on acne vulgaris patients, ethosomal gel containing salicylic acid and clindamycin was shown to be safe and well tolerated with no reports of severe or moderate skin adverse reactions. Interestingly, erythema, burning, pruritus and photosensitivity reactions were lower as compared to commercial topical medications as reported by more than 80% of the participants. Ma et al. [73] investigated the potential toxicity of the paeonol ethosomal system in a histopathologic examination on rats with occlusive treatment for 24 h. The results indicated that the treated skin was normal and identical to the untreated one, no signs of irritation or inflammation were detected, pointing towards the safety of the system.

The tolerability and skin irritation effects of transfersomes were evaluated in several studies. In their study of a sertraline transfersomal system composed of soy lecithin and span 80, Gupta et al. [50] examined the irritant effect of the nanosystem following 7 days of application to guinea pigs. The visually observed results indicated the absence of adverse reactions such as erythema and edema. In another interesting work, Raj et al. [74] examined visually and histopathologically the skin irritant effect of 10 days of application of cytarabine nano-deformable liposomes composed of soy phosphatidylcholine and sodium deoxycholate to rats. Conventional cytarabine solution led to strong erythema. On the other hand, applying the drug in nanovesicles ameliorated this side effect. One possible explanation for this desirable effect is the absence of direct contact between the skin and the drug incorporated inside the nanovesicles.

Vilela et al. [75] studied the in vitro safety of lavender oil-loaded niosomes on adipose tissue-derived stem cells (ASCs) and myometrial cells. The cell viability test indicated no significant difference between the viability of untreated cells and those treated with niosomes indicating the biocompatibility of the carrier.

In three separate clinical studies on healthy volunteers, Paolino et al. [31,63,65] examined the skin tolerability of ammonium glycyrrhizinate incorporated in ethosomes, niosomes, and ultra-deformable liposomes vs. saline and a hydroethanolic solution. The reported results indicated skin tolerability and the absence of toxicity and erythema following the application of the systems.

Overall, the data from numerous in vitro, in vivo and clinical studies carried out on phospholipid and non-phospholipid nanovesicles containing various active molecules indicate their biocompatibility and tolerability on different skin types and cell lines.

## 6. Worldwide Products Designed Based on Nanovesicles as Carriers

The extensive research conducted on nanovesicles for the delivery of active molecules into the skin resulted in several products that were approved in various countries. Products based on phospholipid nanovesicles include Supravir (Trima, Kibbutz Ma’abarot, Israel) an ethosomal cream of acyclovir for the treatment of herpes simplex; Noicellex (Novel Therapeutic Technologies, Herzliya, Israel) an ethosomal cream for reduction of skin cellulite; Skin Genuity (Physonics, Nottingham, UK), an ethosomal cream for reduction of skin cellulite; Lipoduction (Osmotics, New York, NY, USA) an ethosomal lotion for reduction of skin cellulite; Cellulight EF (Hampden Health, Wahroonga, Australia) a cream for reduction of skin cellulite; Decorin Cream (Genome Cosmetics, Bensalem, PA, USA) an anti-aging and skin repair cream; Nanominox (Sinere, Ludwigshafen, Germany) an ethosomal composition for hair growth promotion [1,2].

Commercial products containing niosomes were mostly designed for cosmetic purposes. These include Niosome Plus Lancôme^®^ (Clinchy, France) a foundation for clear and balance skin tone; Niosome Plus perfected age treatment Lancôme^®^ (Clinchy, France) an anti-wrinkle cream; Mayu Niosome Base Cream Laon Cosmetics^®^ (Seoul, Republic of Korea) a whitening and hydrating product; Anti-Age Response Cream Nouvelle-HAS cosmetics^®^ (Varese, Italy) an anti-wrinkle product; Identik Masque Floral Repaire Identik^®^ (Paris, France) a hair repair masque; Indentik Shampooing Floral Repair Identik^®^ (Paris, France) Hair repair shampoo Eusu Niosome Makam Pom Whitening Facial Cream Eusu^®^ (Bangkok, Thailand) a whitening cream; Anne Möller Anti-Fatigue Eye Contour Roll-On Anne Möller^®^ (Barcelona, Spain) an anti-puffiness and moisturizing product [76].

## 7. Conclusions

This review highlights the advances in nanovesicular carriers for improved delivery of active molecules into and through the skin. It covers a large number of publications focusing on their evolution from the classic liposome to more advanced soft vesicles and non-phospholipid systems. It traces the four-decade journey of these carriers and highlights the significant advances made through innovative changes in vesicle composition and properties to overcome the skin barrier.

The numerous in vitro, in vivo, and clinical studies on dermal/transdermal delivery of a wide variety of active molecules, including medical and cosmetic ones, indicate the promising role of these types of carriers. Skin diseases and systemic conditions were successfully treated with active ingredients incorporated in nanovesicles. Moreover, a section is dedicated to the biocompatibility and tolerability of these nanovesicles.

## Figures and Tables

**Table 1:** Differences between various types of nanovesicles for drug delivery into and across the skin.

Type	Vesicles Modifying Agent	Preparation Method	Year	Reference
Liposomes	--	Film formation and hydration	1964	[14]
Phospholipid flexible nanovesicles				
Tranfersomes	Surfactants	Film formation and hydration	1992	[15]
Ultradeformable liposomes	Surfactants	Film formation and hydration	2003	[5]
Phospholipid soft nanovesicles				
Ethosomes	Alcohol	Mixing method	1998	[12]
Glycerosomes	Glycerin	Film formation and hydration	2013	[16]
Tranethosomes	Surfactants and alcohol	Mixing method	2012	[17]
Plurethosomes	Copolymer Pluronic	Mixing method	2021	[18]
Non-phospholipid vesicles				
Niosomes	Non-ionic surfactants	Mixing method; Film forming and hydration	1975	[9]

**Table 2:** In vitro studies on nanovesicular systems for enhanced drug delivery into and across the skin.

Active Molecule	In Vitro Study	Results	Reference
Transfersome			
Methotrexate	Permeation of Methotrexate through porcine skin	Improved skin permeation of Methotrexate by deformable liposomes by 4 folds in comparison with a conventional liposome	[22]
Quercetin	Wound healing assay on HaCat and Ht-144 cells	Enhanced wound healing by Quercetin Ethosomes and Transethosomes	[40]
Ethosome			
Minoxidil	Permeation of Minoxidil through porcine ear skin	Improved skin permeation of Minoxidil from Ethosome by 45 times in comparison to a hydroethanolic solution	[11]
Paclitaxel	Permeation study on human stratum corneum (SC); anti-proliferative effect in squamous carcinoma cells	Significant anti-cancer effect in squamous carcinoma cell line by Paclitaxel Ethosomal system as compared to free drug	[41]
Psoralen	Permeation and penetration study using Franz diffusion cells and excised rat skin	Remarkable skin targeting, producing 2.15 times more psoralen skin deposition than liposomes	[42]
Transethosome			
Fisetin	Penetration and dermatokinetic studies on rat skin by CLSM	Deeper delivery of Rhodamine B up to 70 µm compared to only 30 µm for the hydroalcoholic solution	[38]
Econazole nitrate	Skin permeation and retention studies followed by anti-fungal activity against *C. albicans* fungus	Transethosomal gel exhibited high ex-vivo skin retention 38.75 ± 2.88%, and superior in-vitro antifungal activity in comparison with marketed cream of econazole nitrate.	[43]
Glycerosome			
Diclofenac sodium	Penetration and permeation studies on new-born pig skin	Enhanced skin drug permeation by up to 5 times in comparison with liposome	[16,27,28]
Plumbagin	Permeation study on rat skin	Enhanced skin permeation by more than 6 folds compared to Plumbagin suspension	[44]
Plurethosome			
Mangiferin	Anti-inflammatory effect on 3D skin model	The anti-inflammatory effects of Mangiferin Plurethosome improved by 3 times compared to control	[18]
Niosome			
Tretinoin	Permeation through silicone membrane using Franz cells	High local drug concentration. High trenitoin permeation Best formulation: TritonCG 110-Niosomes	[45]
Ammonium glycyrrhizinate	Release studies of niosomal system embedded in gel matrix	A slower diffusion rate of Ammonium glycyrrhizinate (AG) loaded in Niosomes embedded in the gel matrix as compared to non-embedded vesicles	[31]
Ibuprofen	The effect of niosome composition in the in vitro skin accumulation and transdermal permeation though rat skin	The more lipophilic the surfactant (Tw20/Chol, Tw20/Chol/Chems, Sp60/Chol, Sp60/Chol/Chems), the greater its ability to penetrate the skin	[46]

**Table 3:** In vivo preclinical studies on nanovesicular systems for enhanced drug delivery into and across the skin.

Active Molecule	In Vivo Study	Results	Reference
Transfersome			
Hydrocortisone Dexamethasone	Evaluation of the anti-inflammatory effect in mice with arachidonic-induced edema	Prolonged suppression of the drug-induced oedema nearly 2-fold (to ~24 h per application). The effective dose of dexamethasone delivered with very deformable vesicles into murine skin is reduced >10 times compared with the crème- or lotion-based products.	[49]
Sertraline	Evaluation of the antidepressant activity using the forced swim model test	Transfersomal gel had better antidepressant activity with 0.323 min immobility compared to 2 min in the group treated with the control gel.	[50]
Lycopene	The anti-inflammatory effect of lycopene loaded in transfersomes and ethosomes in mice with anthralin-induced ear swelling.	The results show that lycopene applied from the nanosystems inhibited ear swelling with no significant difference between the systems	[51]
Adapalene	Treatment of acne vulgaris in rats with testosterone-induced acne	Papule count was reduced 3-fold after 4 weeks of the therapy as compared to commercial gel	[52]
Ethosome			
Cannabidiol (CBD)	Skin permeation and organ distribution in mice; evaluation of the reduction in paw thickness in carrageenan-induce edema	Prevention of inflammation and edema in an animal model of Rheumatoid Arthritis by CBD Ethosomal system	[53]
Erythromycin	Antibacterial activity in mice models of deep dermal *S. aureus* infection	Efficient treatment of deep skin bacterial infections in rats by Erythromycin Ethosomes compared to Hydroethanolic solution	[25]
Buspirone	Study on ovariectomized rats with hot flushes	Efficient Management of hot flushes by Buspirone Ethosomes compared to oral treatments. Temperature alleviation to normal values that lasted 3 h)	[4]
Ibuprofen	Measurement of drug plasma concentrations in rats; in vivo antipyretic effect in fevered rats and analgesic effect using tail flick test in mice Anti-pyretic effect of Ibuprofen Ethosomal system.	Temperature alleviation to normal values that lasted at least 12 h versus 7 h after the oral treatment	[2]
Ligustrazine	Pharmacodynamic study on rat model of acute myocardial ischemia	Significant differences in blood viscosity, plasma viscosity, hematocrit, red blood cell aggregation index, as well as deformation index between the ligustrazine ethosomal patch group and ischemic control group (*p* < 0.01). Ligustrazine ethosome patches exhibited a sustained-release property by maintaining stable and sustained blood drug concentration, leading to increased bioavailability, and reduced administration times.	[54]
Transethosome			
Fe-chlorophyllin	Evaluation of the anti-tumor effect in mice	Successful treatment of resistant melanoma without recurrence for 8 months by a combination of the nanovesicles with photodynamic therapy	[26]
Olmesartan medoxomil	Histopathological, pharmacodynamic, and dermatokinetic studies in rats	Controlled blood pressure values for 24 h, with the highest percentage reduction at four hours (35 ± 12%). On the other hand, the oral drug administration led to controlled values for six hours only.	[55]
Asenapine maleate	Pharmacokinetic study in rats	Significant (*p* < 0.05) increase in the bioavailability upon transdermal application of the ethanolic transfersomes compared with oral administration.	[56]
Imiquimod	In vivo skin histological examination in rats; permeation study on rats	Enhanced skin localization by 3 folds compared to commercial product	[57]
Glycerosome			
Paeoniflorin	Permeation experiments through excised rat abdominal skin; in vivo deposition in rat synovium	Glycerosomes modified with Speranskia tuberculata essential oil enhanced the skin deposition of the molecule by three folds compared to conventional glycerosomes	[58]
Cuperron	Permeation study in rat hairless skin; anti-inflammatory effect in carrageenan-induced acute inflammation in rats	Six folds increase in the inhibition of paw edema in comparison with indomethacin commercial product (*p* < 0.05)	[59]
Niosomes			
Meloxicam	Pharmacodynamic study on a rat model of carrageenan-induced paw edema.	Anti-inflammatory effect of Meloxicam niosomes. Paw edema reduced by 2 times in comparison with conventional gel	[60]
Curcumin	Skin irritation test in rats; antinociceptive and anti-inflammatory effect in mice	Curcumin niosomal gels were found to be not irritant. Considerable antinociceptive and anti-inflammatory activities compared to conventional gel	[61]

**Table 4:** Clinical studies on nanovesicular systems for enhanced drug delivery into and across the skin.

Active Molecule	Clinical Study	Results	Reference
Transfersome			
Insulin	Hypoglycemic effect of insulin in a clinical study on healthy volunteers to study	Longer hypoglycemic effect in healthy volunteers of Insulin Transfersome compared to subcutaneous injection of the drug	[5]
Terbinafine	Phase two clinical study with bilateral onychomycosis of the toenail.	Anti-fungal activity within 7 days	[62]
Ammonium glycyrrhizinate	Anti-inflammatory activity of Ammonium glycyrrhizinate Ultra-deformable liposomal system on 8 healthy volunteers	Erythema reduced by 15–30 folds with respect to an aqueous solution	[63]
Ketoprofen	Randomized clinical study conducted on knee osteoarthritis patients	Ketoprofen-containing systems showed significantly higher rates versus the group receiving the empty vehicle	[64]
Ethosome			
Ammonium glycyrrhizinate	Anti-inflammatory activity of Ammonium glycyrrhizinate Ethosomes on 12 healthy volunteers with chemically induced erythema.	The ethosomal system antagonized the appearance of erythema	[65]
Acyclovir	Antiviral effect of Acyclovir Ethosomes in a two-armed, double-blind, randomized clinical study on 40 participants with Herpes labialis	A significant improvement of all the evaluated clinical parameters (crusts formation, loss of crusts, pain)	[66]
Prostaglandin E1	An “in office” pilot clinical study on Prostaglandin E1 Ethosomes for Treatment of Erectile Dysfunction.	Enhanced penile rigidity and prolonged erection duration (10–60 min)	[2]
Clindamycin and Salicylic acid	Treatment of Acne vulgaris by Clindamycin and Salicylic acid ethosomal gel in a clinical study	Improved condition in 71% of the participants	[2]
Niosome			
Ammonium glycyrrhizinate (AG)	Anti-inflammatory activity of Ammonium glycyrrhizinate (AG) Niosomal system on healthy volunteers.	Erythema reduced by 3.4 folds with respect to an aqueous solution	[31]
Methylene blue combined with phototherapy	Four months follow-up clinical study on 40 acne vulgaris patients	The niosomal gel of methylene blue combined with phototherapy showed a significantly higher improvement in inflammation when compared with IPL treatment	[67]
Anthocyanin complex	A randomized placebo-controlled double-blind study on 60 patients (18–60 years old) with oral wounds	AC niosomal gel accelerated wound closure, reduced pain due to oral wounds, and improved the patient’s quality of life as compared to AC gel, triamcinolone gel, and placebo gel.	[68]

## Data Availability

The original contributions presented in the study are included in the article, further inquiries can be directed to the corresponding author.
